# A Comparative Analysis of Clinical and Molecular Factors with the Stage of Cervical Cancer in a Brazilian Cohort

**DOI:** 10.1371/journal.pone.0057810

**Published:** 2013-03-07

**Authors:** Sergio M. Amaro-Filho, Jonathan E. Golub, Gerard J. Nuovo, Cynthia B. Cunha, José E. Levi, Luisa L. Villa, Cecília V. Andrade, Fabio B. Russomano, Aparecida Tristão, Andrea Pires, Alcina F. Nicol

**Affiliations:** 1 Laboratory of Interdisciplinary Medical Research (LIPMED), IOC – Fiocruz, Rio de Janeiro, Brazil; 2 Department of Epidemiology Infectious Diseases, Johns Hopkins Bloomberg School of Public Health, Baltimore, Maryland, United States of America; 3 Comprehensive Cancer Center, Ohio State University, Columbus, Ohio, United States of America; 4 Department of Infectious Disease of Evandro Chagas Clinical Research Instituto, IPEC - Fiocruz, Rio de Janeiro, Brazil; 5 Instituto de Medicina Tropical Laboratório de Virologia – USP, São Paulo, Brazil; 6 Ludwig Institute for Cancer Research – Hospital Alemão Oswaldo Cruz, São Paulo, Brazil; 7 Fernandes Figueira Institute, Fiocruz, Rio de Janeiro, Brazil; 8 Fonte Medicina Diagnostica Laboratory and UFF, Niterói, Brazil; Centro di Riferimento Oncologico, IRCCS National Cancer Institute, Italy

## Abstract

Cell cycle protein expression plays an important role in the pathophysiology of cervical cancer. However, few studies have attempted to correlate the use of these biomarkers with the clinical progression of the tumor.

**Objectives:**

1) To analyze the expression of Ki-67, p53 and p16^INK4a^ in cervical cancer, 2) to correlate the relative expression of these proteins as well as clinical parameters with the stage of disease, and 3) to determine the HPV DNA prevalence and subtype distribution.

**Methods:**

Tissue Micro-Arrays (TMA) from patients with invasive cervical cancer (ICC) and controls were analyzed. HPV DNA detection was done by PCR and in situ hybridization. Ki-67, p53 and p16^INK4a^ were analyzed by immunohistochemistry; clinical data was derived from the chart review.

**Results:**

Advanced tumor stage (III and IV) was strongly associated (p<0.005) with advanced age (>55 years old), with more than four pregnancies and with the lack of formal education. HPV DNA was found in 94.3% of cases with the most prevalent types being HPV16 (67.5%), followed by HPV33 (12.0%) and HPV35 (3.6%). High expression of Ki-67 and p16 was more common in the advanced FIGO stages (p = 0.023). Women with HPV16 tended to be younger (50.9 years; SE 1.9) compared to women with other types (59.9 years; SE 2.8).

**Conclusion:**

We found that Ki-67 and p16 expression were independently associated with the tumor stage. We also noted that about 1/3 of the cervical cancers in this Brazilian cohort were not associated with HPV types directly targeted by the current HPV vaccines.

## Introduction

Despite the promising results achieved in the last decades with the screening of asymptomatic women by Pap smears and more recently with the advent of vaccines against HPV, cervical cancer is still a common disease with about 530,000 new cases and 275,000 deaths per year [1]. The classical management of invasive cervical cancer (ICC) involves evaluating tumor extent which includes tumor size, depth of invasion, microvascular space tumor invasion, spread to regional lymph nodes, and grade of differentiation. The treatment of cervical cancer is predicated on the evaluation of the clinical stage of tumor according to the classification of the International Federation of Gynecology and Obstetrics (FIGO). For early-stages (FIGO I-IIA) either surgery or radiotherapy (RT) is employed, whereas for late-stages (FIGO IIB-IV) chemotherapy is indicated [2]. However, clinical staging has certain limitations due to variables such as inter-observer variability. Discrepancies have been reported in up to 25% of cases in early stage disease and 65–90% in advanced (≥IIB) disease [3], [4].

Imaging technologies, such as computed tomography and ultrasonography, have been adopted to improve the clinical staging accuracy of cervical cancer. However, some studies have reported low sensitivity and high false-negative results with these methods [2]. Thus, new predictive markers are needed to identify patients with high risk of relapse, poorer prognosis, and to optimize disease management, especially in early invasive cervical cancer (ICC).

Persistent infection with certain human papillomavirus types (especially types 16 and 18) has been well documented as a necessary co-factor for cervical cancer development. The high risk HPV types are able to actuate the complex pathways that ultimately leads to an invasive cancer, in part, due to the ability of E6 and E7 viral oncoproteins to drive cells into S-phase [5]. E7 associates with retinoblastoma protein (pRb) that is a tumor suppressor protein related to several major cancers. pRb prevents the cell from replicating damaged DNA by preventing its progression along the cell cycle through G1 (first gap phase) into S (synthesis phase) [6]–[9] and is involved in preventing excessive cell growth by inhibiting cell cycle progression until the cell is ready to divide when pRb binds and inhibits the E2F family of transcription factors [10], [11]. After E7 and pRb association, E2F proteins are able to subsequently transactivates cellular cyclin-dependet kinases (CDKs) proteins, required for viral DNA replication, that can lead to cancer [6]. Futhermore, E7 is also capable of interacting with other proteins involved in cell proliferation such as histone deacetylases, components of the AP1 transcription complex and cyclin-dependent kinase inhibitors including p21 and p27 [12]. E6 is able to mediate p53 ubiquitination and degradation. This, in turn, reduces the effectiveness of the cellular DNA damage response and allows the accumulation of secondary mutations which, in turn, increases the risk of cancer formation [5].

Antigen KI-67 also known as Ki-67 or MKI67 is a nuclear protein that in humans is encoded by the MKI67 gene and is strictly associated with cell proliferation. Ki-67 protein is present during all active phases of the cell cycle (G1, S, G2, and mitosis), but is absent from resting cells. It has been found to be a reliable predictive factor for tumor development. Thus, the Ki-67 proliferation index, which reflects the percentage of tumor cells that are actively proliferating, is a commonly used marker in diagnostic pathology for differentiating benign from malignant tumors [13]–[15]. P16^INK4a^ is a cyclin-dependent kinase inhibitor which acts as a tumor suppressor by binding to cyclin-dependent kinases as CDK4 and CDK6, preventing the phosphorylation and subsequent inactivation of the retinoblastoma protein (pRB), in humans is encoded by the CDKN2A gene and plays an important role in regulating the cell cycle. This gene generates several transcript variants that differ in their first exons. Recently one study found several novel genes to be differentially expressed in cervical cancer and was overexpressed in cervical cancers, confirmed by immunohistochemistry such as MMP3, UBE2C and p16 protein [16]. Diffuse p16^INK4a^ expression has been related to high-risk HPV (HR-HPV) infection, especially in high grade cervical intraepithelial neoplasia and cancer [17]–[19].

The protein P53, known as protein 53 or tumor protein 53, is a tumor suppressor protein where it regulates the cell cycle and, thus, functions as a tumor suppressor that is involved in preventing cancer. P53 has an important role in conserving stability by preventing genome mutation. In humans, p53 is encoded by the TP53 gene located on the short arm of chromosome 17. In unstressed cells, p53 levels are kept low through a continuous degradation of p53 however If the TP53 gene is damaged, tumor suppression is severely reduced [20]. The TP53 gene can also be damaged in cells by mutagens increasing the likelihood that the cell will begin decontrolled division. More than 50 percent of human tumors contain a mutation or deletion of the TP53 gene. In cervical cancer, the E6 HPV oncoprotein binds to p53 and promotes its degradation by the ubiquitin proteolysis pathway [21]–[24]. Some pathogens, as HPV can also affect the p53 proteins expression. Conflicting results have been published concerning p53 expression, some studies has showing a positive correlation of p53 with high grade lesions and cervical cancer, whereas others declare no significant associations [21], [25].

The present study was performed to examine the hypothesis that the proteins involved in cell cycle Ki-67, p53 and p16^INK4a^ are overexpressed in the uterine cervix of patients with cervical cancer and, more specifically, to evaluate the correlation between these biomarkers with the FIGO stage and the HPV genotype. Furthermore, socio-demographic, behavioral and clinical characteristics from the study population were also analyzed, therefore our main aims were 1) To analyze the expression of Ki-67, p53 and p16^INK4a^ in cervical cancer 2) to search for a differential expression that can assist in the assessment of clinical tumor staging according to FIGO classification, and 3) to determine the HPV DNA genotype distribution in this population.

## Materials and Methods

### Tissue samples and data collection

The study material consisted of 130 samples randomly selected from the archives of the Fernandes Figueira Institute, Oswaldo Cruz Foundation, Rio de Janeiro, Brazil. Samples from 87 patients obtained by cervical biopsy or conization between 2003 and 2008 were histopathologically confirmed by a surgical pathologist as invasive cervical cancer. Forty-three patients undergoing hysterectomy for benign leiomyomata disease used as controls.

Sociodemographic and health data were obtained from individual patient charts. The following data were collected: age, history and pack years of tobacco smoking, history of alcohol consumption, contraceptives (hormonal), age of first sexual intercourse, numbers of pregnancies, education level, HIV-1 serology and FIGO stage. The Institutional Review Board (IRB) from Oswaldo Cruz Foundation (Fiocruz), (CAE 0024.0.011.000-09-,Rio de Janeiro, Brazil) approved the use of written consent in this study.

#### Tissue Micro-Array (TMA) block construction

The Tissue Micro-Array blocks were constructed as described by Pires et al, 2006 [26]. Briefly, all hematoxylin-eosin (HE) slides were re-examined by a second experienced pathologist and two morphologic representative fields of invasive cervical cancer were chosen and encircled with a marker pen. Two cores from each case were punched out from the donor blocks. The corresponding H&E slides were overlaid with a custom-built 16 gauge Becton-Dickinson PrecisionGlide® hypodermic needle (1,1 mm^2^ area). Afterwards, cores were attached by double-side adhesive tape on a computer-generated paper grid affording alignment on the block mould, which was then filled with liquid paraffin. Three µm thick sections were obtained from an optical standard rotator microtome (Leica, Bensheim, Germany). Each block provided 40–50 slides; only samples showing the original lesion were used. A total of two TMA blocks were constructed, one with cervical cancer biopsies and the other with controls in which the absence of cervical intraepithelial neoplasia (CIN) was confirmed by review of the hematoxylin and eosin stained slide.

#### DNA extraction

From each paraffin embedded cervical biopsy, 4 slices of 5 µm were cut. Briefly, after dewaxing with xylene at 48°C for 2 hours, ethanol (100%, 70% and 50%) was added to remove residual xylene, followed by rehydration and centrifugation at 12,000 rpm per 15 min in each step. The pellet was resuspended in solution with 300 µL of proteinase K (100 µg/mL) and 10% SDS during 48 hours at 48°C. Five microliters of proteinase K (200 µg/mL) were added after 24 hours. DNA was isolated by a phenol extraction containing 300 µL of phenol/chloroform/isoamyl alcohol (25/24/1) followed by centrifugation (12,000 rpm per 10 min) to obtain the aqueous phase. After repeating these purification steps twice, the DNA was precipitated at 20°C for 24 hours with 100 µL sodium acetate (final concentration 7.5 M) and twice the aqueous phase volume of 100% ethanol. The DNA pellet was collected by centrifugation for 15 min at 12,000 rpm at 4°C, washed with 70% ethanol and resuspended in 50 µL of TE buffer (10 mM Tris base, 1 mM EDTA, pH 7.5). The DNA quantity and quality was analyzed by NanoDrop (Thermo Fisher Scientific, Wilmington, DE, USA).

#### PCR amplification and HPV genotyping

Amplification of the HPV L1 consensus region was performed with generic primers GP5+ and GP6+ (synthesized by Invitrogen, Sao Paulo, SP, Brazil) to generate a PCR product of approximately 150 bp as previously described (synthesized by Invitroge*n*, Sao Paulo, SP, Brazil; forward primer sequence: GP5+ *TTTGTTACTGTGGTAGATACTAC*; reverse primer sequence GP6+ *GAAAAATAAACTGTAAATCATATTC*) [27], [28]. Negative samples with these primers were subjected to nested PCR using a first round amplification with primers PGMY09 and PGMY11, previously described (synthesized by Invitrogen, Sao Paulo, SP, Brazil; forward primer sequence: PGMY11A *GCACAGGGACATAACAATGG*, PGMY11B *GCGCAGGGCCACAATAATGG*, PGMY11C *GCACAGGGACATAATAATGG*, PGMY11D *GCCCAGGGCCACAACAATGG*, PGMY11E *GCTCAGGGTTTAAACAATGG*; reverse primer sequence: PGMY09F *CGTCCCAAAGGAAACTGATC*, PGMY09G *CGACCTAAAGGAAACTGATC*, PGMY09H *CGTCCAAAAGGAAACTGATC*, PGMY09I *GCCAAGGGGAAACTGATC*, PGMY09J *CGTCCCAAAGGATACTGATC*, PGMY09K *CGTCCAAGGGGATACTGATC*, PGMY09L *CGACCTAAAGGGAATTGATC*, PGMY09M *CGACCTAGTGGAAATTGATC*, PGMY09N *CGACCAAGGGGATATTGATC*, PGMY09P *GCCCAACGGAAACTGATC*, PGMY09Q *CGACCCAAGGGAAACTGGTC*, PGMY09R *CGTCCTAAAGGAAACTGGTC*, HMB01 *GCGACCCAATGCAAATTGGT*) [29]. Every PCR assay included as a positive control Hela cellular DNA (HPV18 DNA) and a negative template control. The integrity of specimen DNA was verified by amplification of 110 bp fragment of β-globin gene with primers PC03 and PC04. The amplifications were carried out in a GeneAmp PCR System 9700 thermal cycler (Applied Biosystems), and all PCR products were analyzed by electrophoresis in 1.5% agarose gel.

A final reaction volume of 75 µL was purified by the GF-1 DNA Recovery Kit (Vivantis, Oceanside, CA, USA), in order to genotype. For sequencing a solution containing 3.2 pmol of each primer (GP5+ or GP6+), 5–10 ng of PCR product, 2.5 µL Big Dye Terminator v 3.1 Cycle Sequencing Kit (part number 4337455; Applied Biosystems, Foster City, CA, USA) and MilliQ water completing for final volume of 10 µL was prepared. The mixtures were analyzed on an ABI Prism 3730 Genetic Analyzer (Applied Biosystems) available at the DNA PDTIS/Fiocruz Platform [30]. Alignments were obtained directly from on-line Blastn server or phylogenetically analyzed by the software MEGA5 [31]. Cases not amplified by PCR or unable to be sequenced where checked for HPV DNA by means of the INNO-LiPA HPV Genotyping v2 (Innogenetics, Gent, Belgium). Three equivocal cases were also analyzed by the PapilloCheck Kit (Greiner Bio-One, Frickenhausen, Germany).

#### Immunohistochemistry (IHC)

IHC reactions were performed on TMA silane-coated slides (Sigma, St. Louis, MO, USA), as previously described [32]. Briefly, slides were dewaxed in xylene, rehydrated through a graded ethanol series, washed with distilled water and then treated in solution with methanol containing 0.3% hydrogen peroxide for 10 min to eliminate endogenous peroxidase activity. Antigen retrieval for all immunohistochemistry was performed by boiling the tissues with the Target Retrieval Solution (Dako, Carpinteria, CA, USA). Non-specific antibody binding was inhibited by incubating sections with serum-free protein block (1% BSA). Tissue sections were sequentially incubated overnight in a humidified chamber at 4°C temperature with the primary specific antibodies (dilutions): monoclonal antibody anti-Ki67 (ready to use – Dako, Carpinteria, CA, USA), monoclonal antibody anti-p53 (1/10 – Santa Cruz, Biotechnology, Inc-CA-USA) and monoclonal antibody anti-p16^ink4a^ (1/250 – Santa Cruz, Biotechnology, Inc-CA-USA). A secondary biotinylated multilink antibody was applied for 30 min followed by streptavidin-peroxidase for 30 min. The enzymatic reaction was developed in a solution of 3.3′-diaminobenzidine (DAKO, Glostrup, Denmark) exposure for 5 min, except for p53 where the FastRed chromogen was used (Sigma Chemical Co., St. Louis, MO, USA). Slides were counterstained with hematoxylin for 1 min, washed in distilled water, dehydrated in graded ethanol, cleared with xylene and permanently mounted in Entellan (Merck, Darmstadt, Germany).

### Assessment of IHC slides and cell counting

Two independent observers reviewed and quantified the expression of all immunohistochemical stains. The Ki-67 and p53 stain included nuclear staining only. Two representative areas of each core were manually counted by the observers and for Ki-67, classified as: negative, less than 25% positive cancer cell nuclei, 25–50% positive or more than 50% positive. For p53 the scoring system was as follows: negative, less or equal to 5% positive cancer cell nuclei, 5–25% positive and greater than 25%. Immunohistochemical expression for p16^INK4a^ was quantified according to presence (positive or negative), cellular location (nuclear and cytoplasmic), staining intensity (weak, moderate and strong) and dispersion pattern (diffuse or focal).

#### Statistical Analysis

Data were analyzed by means of the STATA/SE 10.1 software. Statistical comparisons were performed using the parametrical Student t test for continuous variables as well as the chi-square test and the Fisher's exact test for categorical variables. A Test for trend across ordered groups (nptrend) was incorporated to observe if the biomarkers were following a linear trend expression for the FIGO stage. The Univariated logistic regression was applied to verify the association between the demographic, behavioral and clinical factors with the ICC and control groups. Sensitivity (probability of a true positive), specificity (probability of a true negative) and Youden's Index (YI) (YI = sensitivity+specificity−1), as a measure of overall diagnostic effectiveness, were calculated for consensus diagnoses of FIGO III–IV and II–IV. All p values are two-sided; p-values<0.05 were considered statistically significant.

## Results

### Study population characteristics analysis

This study included 130 women (average age 51.1±13.1 years old; range 24–88). In order to compare clinical variables and FIGO stage, socio-demographic, behavioral and clinical characteristics were gathered from individual patient charts. However, some data were not available for all patients as following (N - %): age (6–4.6%), education level (41–31.5%), tobacco smoking (41–31.5%), alcohol consumption (43–33.1%), contraceptives (48–36.9), first sexual intercourse (53–40.8%), numbers of pregnancies (33–25.4%), HIV-1 status (86–66.2%) and FIGO stage (17–19.5%).

### Associations of histopathology and FIGO stage with socio-demographic and clinical characteristics

In this population, by univariated logistic regression analysis, it was noted that tobacco smoking, alcohol consumption, contraceptives (oral contraceptive pills), age of first sexual intercourse, and HIV-1 status were unrelated to cervical cancer. However, there was a statistically significant correlation between the cervical cancer and control groups in the following categories: patients older than 55 (OR = 16.6; CI95% = 3.5–79.2; p<0.001), women without formal education (OR = 8.0; CI95% = 1.5–43.4; p = 0.016) and women with more than four pregnancies (OR = 7.2; CI95% = 2.4–21.5; p = 0.001) (Table 1). Further, among the patients with cervical cancer, there was a statistically significant association between increased numbers of pregnancies (3 or more) as well as a lack of formal education when comparing FIGO stage I to FIGO stages II–IV (p = 0.005).

**Table 1:** Association between the patient information with the tissue morphology and the FIGO stage.

Characteristics	Control (N = 43)	Cancer (N = 87)	OR (CI 95%)	*P*	FIGO stage			P*
					I	II	III and IV	
**Age. years (N = 124)**								
<45	18 (47.4)	19 (24.7)	1		10 (52.6)	4 (17.4)	2 (8.3)	**0.006**
45–55	18 (47.4)	23 (29.9)	1.2 (0.5–2.9)	0.675	4 (21.0)	9 (39.1)	6 (25.0)	**np_trend_ = 0.001**
>55	2 (5.2)	35 (45.4)	16.6 (3.5–79.2)	<0.001	5 (26.3)	10 (43.5)	16 (66.7)	
**Education level (N = 89)**								0.256
No formal education	2 (5.9)	16 (29.1)	8.0 (1.5–43.4)	0.016	1 (8.3)	7 (38.9)	7 (43.7)	**np_trend_ = 0.039**
≤Basic Eductation	21 (61.8)	28 (50.9)	1.3 (0.5–3.6)	0.576	8 (66.7)	8 (44.4)	8 (50.0)	
≥High School	11 (32.3)	11 (20.0)	1		3 (25.0)	3 (16.7)	1 (6.3)	
**Tobacco smoking (N = 89)**								0.791
Yes	15 (40.5)	26 (50.0)	1.5 (0.6–3.4)	0.378	6 (46.2)	11 (55.0)	6 (60.0)	
No	22 (59.5)	26 (50.0)	1		7 (53.8)	9 (45.0)	4 (40.0)	
**Alcohol consumption (N = 87)**								0.479
Yes	12 (32.4)	12 (24.0)	0.6 (0.2–1.6)	0.386	4 (33.3)	3 (15.0)	2 (22.2)	
No	25 (67.6)	38 (76.0)	1		8 (66.7)	17 (85.0)	7 (77.8)	
**Contraceptives (hormonal) (N = 82)**								0.459
Yes	9 (25.7)	7 (14.9)	0.5 (0.2–1.5)	0.226	2 (18.2)	2 (9.5)	2 (28.6)	
No	26 (74.3)	40 (85.1)	1		9 (81.8)	19 (90.5)	5 (71.4)	
**First sexual intercourse (N = 77)**								0.752
≤17 anos	11 (33.3)	23 (52.3)	1		4 (40.0)	9 (52.9)	5 (55.6)	
>17 anos	22 (66.7)	21 (47.7)	0.9 (0.8–1.0)	0.091	6 (60.0)	8 (47.1)	4 (44.4)	
**Numbers of pregnancies (N = 97)**								**0.049**
No one					4 (26.7)	-	2 (12.5)	np_trend_ = 0.056
1–2	18 (50.0)	10 (18.5)	1		4 (26.7)	3 (14.3)	1 (6.3)	
3–4	16 (44.4)	18 (33.3)	2.2 (0.7–6.8)	0.186	1 (6.6)	7 (33.3)	2 (12.5)	
>4	2 (5.6)	26 (48.2)	7.2 (2.4–21.5)	0.001	6 (40.0)	11 (52.4)	11 (68.7)	
**HIV-1 status (N = 44)**								0.082
Yes	1 (10.0)	3 (8.8)	0.9 (0.0–9.4)	0.909	3 (25.0)	-	-	
No	9 (90.0)	31 (91.2)	1		9 (75.0)	7 (100.0)	11 (100.0)	

### HPV prevalence and distribution

Two of the 43 control samples yielded unsatisfactory results for DNA extraction and were excluded from HPV DNA prevalence analyzes. Three samples negative by PCR with GP5+/6+ were positive for viral DNA by a nested PCR using PGMY09/11 primers. The total prevalence of HPV DNA in the control and cervical cancer groups was 29.3% (12/41) and 95.4% (83/87), respectively. The HPV genotype distribution in the control and in the cervical cancer groups is shown in the table 2. Infections with more than two types of HPV in a single sample were rare; 2/92 HPV positive samples showed multiple infections. In the control group, HPV type 16 revealed the highest prevalence (72.7%) followed by types 35, 67 and 6 (9.1% for each). In women with cervical cancer, HPV type 16 was also the most prevalent (67.5%), followed by type 33 (12.0%) and type 35 (3.6%). HPV type 58 and 6 were found in two cases (2.4%) while HPV 18, 31, 52, 67, 70, 69 and 30 were found in only 1 case (1.2%). Women infected with HPV16 exhibited a statistical significant association (p = 0.012) with younger average (50.9; SE 1.9) compared to women with other types (59.9; SE 2.8).

**Table 2:** Distribution of human papillomavirus (HPV) genotypes in single and multiple infections.

HPV type	Control N (%)	Câncer N (%)
**Single Infection**		
High-risk		
16	8 (66.6)	56 (67.5)
33	-	10 (12.0)
35	1 (8.3)	3 (3.6)
58	-	2 (2.4)
18	-	1 (1.2)
31	-	1 (1.2)
52	-	1 (1.2)
Low-risk		
67	1 (8.3)	1 (1.2)
6	1 (8.3)	2 (2.4)
70	-	1 (1.2)
Indeterminated risk		
69	-	1 (1.2)
30	-	1 (1.2)
**Multiple Infection**		
16+52	-	1 (1.2)
16+33+54+52	-	1 (1.2)
**Not identified** *	1(8.3)	2 (2.4)
Total	12 (100)	83 (100)

* HPV DNA positive samples by PCR technique but unable to genotype.

### Protein expression analysis

The relative degree of protein expression as determined by immunohistochemistry was correlated with the FIGO stage and to HPV genotype in the HPV positive cervical cancer cases.

### Ki-67 expression evaluation

**Figure 1:**
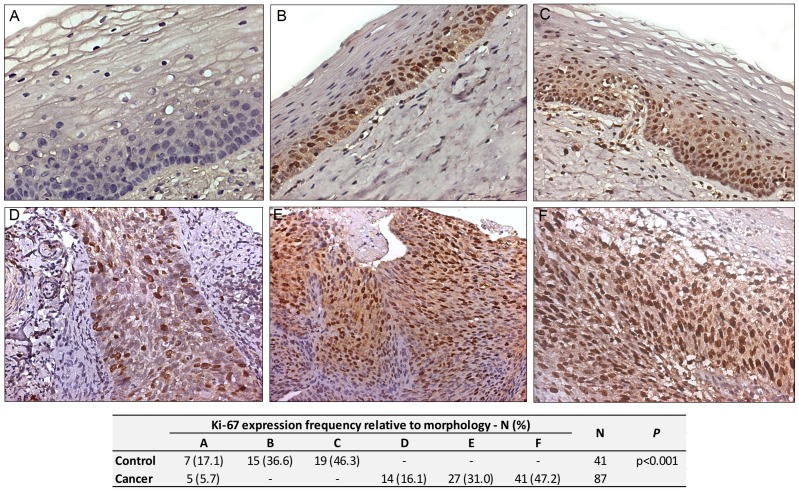
Ki-67 expression. A, B and C: control epithelium; D, E and F: invasive cervical cancer epithelium. (A) Negative; (B) Strict positive stain in the basal layer; (C) positive stain in the basal and intermediate layer; (D) less than 25% of positive cells all over the epithelium; (E) 25–50% of positive cells all over the epithelium; (F) more than 50% of positive cells all over the epithelium (DAB, brown stain; Mag. 40×).

The Ki-67 staining was nuclear and present in 34 (82.9%) of the controls and 82 (94.3%) of the cervical cancer cases. However, there was a marked difference in the distribution pattern of Ki-67 in the cervical cancer versus normal cervical epithelia. Specifically, the Ki-67 positive cells were confined to the basal epithelial layer in the normal cervical tissue whereas, in the cervical cancer specimens, Ki-67 expression was diffuse and present in the majority of cancer cells from the basal aspect to the surface of the malignant tumor. In order to differentiate and quantify the expression pattern between cervical cancer samples and the control, Ki-67 expression was assigned letters (A, B, C, D and F). “A” referred to no Ki-67 signal, “B” denoted positive cells present exclusively in the basal/parabasal epithelium, “C” represented positive cell evidence in the basal/parabasal and intermediate epithelium, “D” referred to a signal from the base to the surface of the epithelia with less than 25% of positive nucleus, “E” referred to a signal from the base to the surface of the epithelia with 25% to 50% of positive nucleus and “F” was associated to a signal from the base to the surface of the epithelia with greater than 50% of positive nucleus (Figure 1). As is evident in Table 3, there was a significant increase trend of Ki-67 expression with an increased FIGO stage (np_trend_ = 0.008) (Table 3).

**Table 3:** Association between the Ki-67 expression frequencies with the FIGO stage.

FIGO Stage	Ki-67 Expression - N (%)				N	*P*
	Negative	<25%	25–50%	>50%		
I	4 (21.0)	6 (31.6)	3 (15.8)	6 (31.6)	19	0.053
II	1 (3.8)	3 (11.6)	9 (34.6)	13 (50.0)	26	np_trend_ = 0.008
III/IV	-	3 (12.0)	7 (28.0)	15 (60.0)	25	

### P16^INK4a^ expression evaluation

**Figure 2:**
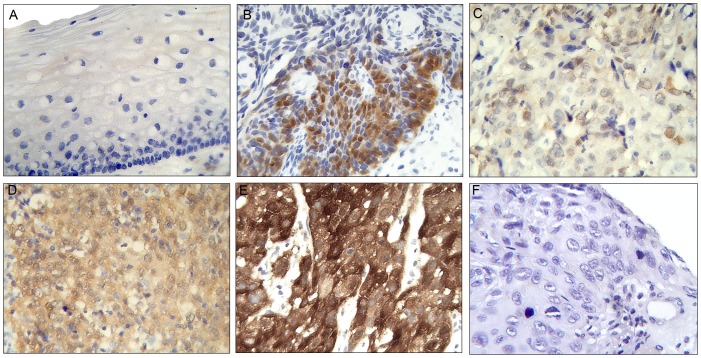
P16^ink4a^ expression. A: control epithelium; B, C, D, E and F: invasive cervical cancer epithelium. (A) Negative; (B) Focal moderate nuclear and cytoplasmatic expression; (C) Diffuse weak nuclear and cytoplasmatic expression; (D) Diffuse moderate cytoplasmatic expression; (E) Diffuse strong nuclear and cytoplasmatic expression; (F) Negative (DAB, brown stain; Mag. 40×).

Different patterns of p16^INK4a^ signal were observed based on cellular location (nuclear and cytoplasmatic), staining intensity (weak, moderate and strong) and dispersion pattern (diffuse or focal). Only 5 (11.6%) of the controls were p16^INK4a^ positive, in contrast, 83 (95.4%) of the cervical cancer tissues were positive (Figure 2). A diffuse pattern of p16 expression was statistically (p<0.001) associated in general with cervical cancer (85.5%) cases versus the controls. Cases presenting a moderate p16^INK4a^ expression were more common in the advanced FIGO stages III–IV (56.5%) while weak expression was associated with early FIGO stage I (66.7%) (p = 0.023) (Table 4). No association was observed between the cellular location pattern of p16^INK4a^ staining and either the tumor grade or FIGO stage (p = 0.152 and p = 0.183, respectively).

**Table 4:** Association between p16 expression patterns with the tissue morphology and the FIGO stage.

	Patterns expression of p16^INK4a^ - N (%)											
	Positive	Negative	N	*P*	Intensity			*P*	Dispersion		N	*P*
					Weak	Moderate	Strong		Diffuse	Focal		
**Morphology**				**<0.001**				1.000				**<0.001**
Control	5 (11.6)	38 (88.4)	43		2 (40.0)	2 (40.0)	1 (20.0)		-	5 (100.0)	5	
Cancer	83 (95.4)	4 (4.6)	87		29 (34.9)	29 (34.9)	25 (30.1)		71 (85.5)	12 (14.5)	83	
**FIGO stage**												
I	18 (94.7)	1 (5.3)	19	0.832	12 (66.7)	3 (16.7)	3 (16.7)	**0.023**	13 (72.2)	5 (27.8)	18	0.213
II	25 (96.1)	1 (3.8)	26		9 (36.0)	7 (28.0)	9 (36.0)	**0.041** *	23 (92.0)	2 (8.0)	25	
III/IV	23 (92.0)	2 (8.0)	25		5 (21.7)	13 (56.5)	5 (21.7)		20 (86.9)	3 (13.0)	23	

* Test for trend across ordered groups (np_trend_) was used to analyze more than two subgroups for the others were used Fisher's exact.

### P53 expression evaluation

**Figure 3:**
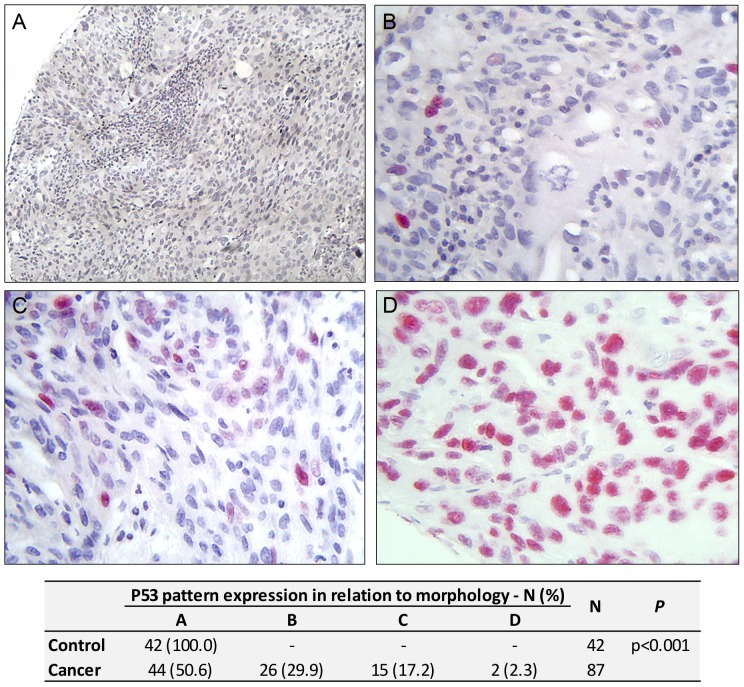
P53 expression. A, B, C and D: invasive cervical cancer epithelium. (A) negative; (B) less than 5% nuclear stain positivity; (C) 5% to 25% nuclear stain positivity; (D) higher than 25% (FastRed, red stain; Mag 40×, Fig A Mag.20×).

Positive p53 expression was detected in 43 of the 87 (49.4%) cervical cancer cases and negative in all the control cases. p53 was expressed exclusively in the nuclear compartment in all positive cases. Immunostaining of less than 5%, 5–25% and greater than 25% of positive cancer cell nuclei was observed in 26 (29.9%), 15 (17.2%) and 2 (2.3%), respectively of cervical cancers (Figure 3).

### Ki-67, p16^INK4a^ and p53 Clinical Performance

We also examined the clinical performance (sensitivity, specificity and Youden's Index) of different positive endpoints for p16^INK4a^, Ki-67 and p53 staining, and the markers combined, in relation to the consensus diagnoses of FIGO III–IV (FIGO III+) and FIGO II–IV (FIGO II+). Combining the positive intensity endpoint for p16^INK4a^ staining with Ki-67 and p53, staining increased the specificity and accuracy for FIGO III+ and FIGO II+ detection (Table 5). The highest sensitivity and specificity were observed in the advanced stages (FIGO II+) by Youden's index; adapting a cutoff of more than 25% of cells expressing Ki-67 and p16^INK4a^ with moderate or strong intensity 93.5%, 100% and 93.5%.

**Table 5:** Clinical performance of p16^INK4a^, Ki-67 and p53 immunostaining in relationship to FIGO stage III or more severe (FIGO III+) and FIGO II+.

	FIGO III+			FIGO II+		
Cutpoint	Se (%)	Sp (%)	YI (%)	Se (%)	Sp (%)	YI (%)
p16 moderate/strong	76,2	46,7	22,9	70,8	66,7	37,5
Ki67 >25%	90,9	31,9	22,8	86,0	52,6	38,6
Ki67 >50%	41,9	57,4	−0,6	54,0	68,4	22,4
Ki67 >75%	22,7	74,5	−2,8	24,0	73,7	−2,3
p53 >5%	50,0	72,0	22,0	30,8	57,1	−12,1
p53 >5% and Ki67 >25%	66,7	70,0	36,7	53,3	81,8	35,2
p53 >5% and Ki67 >50%	27,3	83,3	10,6	24,1	91,7	15,8
p53 >5% and Ki67 >75%	11,8	96,7	8,4	8,3	100,0	8,3
p16 moderate/strong and p53 >5%	100,0	50,0	50,0	30,0	90,9	20,9
**p16 moderate/strong and Ki67 >25%**	93,8	26,3	20,1	**93,5**	**100,0**	**93,5**
p16 moderate/strong and Ki67 >50%	76,9	41,2	18,1	69,0	100,0	69,0
p16 moderate/strong and Ki67 >75%	61,5	93,3	54,9	64,5	100,0	64,5

Se = sensitivity, Sp = specificity, YI = Youden's Index (Sp+Se−100%).

* Highlighted with bold font are the cut point combinations of p16^INK4a^ and Ki-67 that are most accurate (highest Youden's Index) for consensus diagnosis of FIGO II+.

### Relationship of HPV Genotypes to Immunohistochemical Findings

We examined whether HPV 16 or certain combinations of HPV genotypes could influence the expression of the biomarkers analyzed in the ICC samples. High expression of Ki-67 was associated with other HPV types, compared with HPV 16 or when segregated in different groups (HPV 16 single infection, other high-risk, low-risk and indeterminate, and multiple infections) as displayed in table 6. No relationship was observed between p16^INK4a^ expression and HPV group genotypes. There was no significant correlation with p53 expression and HPV genotype (Table 6).

**Table 6:** Correlation between positive expression of biomarkers and groups of HPV genotypes.

HPV type in cancer	Ki-67 Expression - N (%)				p16 Expression - N (%)			p53 Expression - N (%)		
	Negative	<25%	>25%	*P*	Negative	Positive	*P*	Negative	Positive	*P*
HPV 16*	5 (8.6)	12 (20.7)	41 (70.7)	**0.007**	2 (3.5)	56 (96.5)	0.334	29 (50.0)	29 (50.0)	0.460
Other types	-	-	24 (100.0)		1 (4.4)	22 (95.6)		13 (54.2)	11 (45.8)	
HPV 16 single	5 (8.9)	10 (17.9)	41 (73.2)	**0.016**	2 (3.6)	54 (96.4)	0.061	27 (48.2)	29 (51.8)	0.078
Other high-risk	-	-	18 (100.0)		-	18 (100.0)		12 (66.7)	6 (33.3)	
Low-risk/Indeterminated	-	-	6 (100.0)		2 (33.3)	4 (66.7)		1 (16.7.0)	5 (83.3)	
Multiple infections	-	2 (100.0)	-		-	2 (100.0)		2 (100.0)	-	

* single and co-infected samples with HPV16.

## Discussion

We observed an increasing expression of Ki-67, p53 and p16^INK4a^ in invasive cancer specimens compared to normal controls. Since Ki-67 is a classical proliferation marker and has been used to differentiate benign versus malignant tumors at various sites, the strong correlation of Ki-67 and cervical malignancy was expected [33]. Ki-67 was expressed in all layers of the squamous epithelium with invasive cancer. It was also found in normal tissues, however, restricted primarily to the parabasal layer region. These data are in agreement with several studies, where Ki-67 has exhibited high rates of specificity and sensitivity as a biomarker for cervical intraepithelial neoplasia [33], [34].

The p53 protein did not show labeling in normal epithelium. In the invasive cervical cancer specimens we found that 49.4% (43/87) were positive, though showing expression in far fewer nuclei of the carcinoma cells when compared to the expression pattern of other markers. The literature describes conflicting data for p53 expression in cervical cancer, with rates ranging from very low percentages to 62.0% of cancer cells [21], [25]. Although the basis of this marked disparity of results is unclear, it may relate to different causes including different tissue fixation, as well as the antigen retrieval methods and adopted cutting points [35]. It is also known that in many types of human cancer, the p53 is overexpressed as a result of mutations which modify their transcriptional activity, that may, in turn, affect other regulatory proteins for example, MDM2 (also called HDM2 for the human protein). However, in the case of cervical cancer, it appears to be different, since the E6 protein from HPV high risk is associated with the ubiquitination of p53 and subsequent degradation by the proteasome. Nevertheless this does not preclude the possibility that some cervical carcinomas exhibit point mutations of p53 [36]. Our data regarding p53 expression is consistent with previous studies such as by Bahnassy et al. (2007) and Skomedal et al. (1999) who found, respectively, 44.2% (19/43) and 55.4% (41/74) of invasive cervical cancer specimens positive for p53 and in both studies, no case was positive in the control specimens [33], [37].

Regarding p16^INK4a^, several studies have documented overexpression, with a diffuse and strong pattern, not only in high-grade cervical intraepithelial lesions but also with invasive cancer compared to the normal specimens [13], [18], [33]. Our study confirms this increase in the expression in invasive cancer compared to the controls samples. We found 95.4% (83/87) of the invasive cancer were positive for p16^INK4a^ compared to 11.6% (5/43) of the controls. Few papers have observed p16^INK4a^ expression in normal tissues [36], [38], [39]. Still, it is well established that non dysplastic epithelial cells can express p16^INK4a^, for example, under certain physiological conditions, such as the shortening of telomeres in older tissues. Here, the expression of p16^INK4a^ immediately induces cell cycle arrest and may ultimately induce apoptosis with the end results being a focal pattern with weak intensity [40].

Regarding the clinical stage of cervical cancer, our data indicate a statically confirmed correlation between high expression of Ki-67 (>50% of the cancer cells) and increased stage of the disease. One study described an association of the Ki-67 proliferation index with clinical parameters including tumor size, lymph node invasion and disease-free interval in women with stage IB cervical cancer [41]. Bahnassy et al. (2007) observed a statistically significant increase in the number of Ki-67 positive cases of 72.2% (26/36) in stage III–IV, compared to 27.8% (10/36) in stages I–II. However, Ancuta et al. (2009) found no statistically significant correlation between Ki-67 and clinical stage IB and IIB [16].

Our study observed no significant linear association in the expression of p53 related to clinical staging, which corroborates the studies from Tjalma *et al.* (2001) and Skomedal *et al.* (1999) [35], [37]. However, Bahnassy *et al.* (2007) reported a statistically significant number of p53 positive cases in the group with stage III–IV in relation to I–II. For p16^INK4a^, we also did not observe a linear expression, which reinforces the results of Bahnassy *et al.* (2007), [33].

Further, we analyzed whether there were differences between the biomarker expression and the HPV types. Assuming that HPV16 is the most oncogenic HPV type and, therefore, more prevalent in cervical cancer, we divided the biomarkers into two groups: one group with only HPV16 positive cases and the other with the remaining HPV types. Our data indicated an increased expression of Ki-67 in the cervical samples with the other HPV types compared to HPV16.

The p16^INK4a^ protein is a CDK inhibitor, increased among cervical cancer cases which are directly related to expression of the oncogene HPV E7. Abnormal amounts of E7 in basal cells disrupts the binding of pRB, resulting in increased levels of E2F the transcription factor activator of the cell cycle, which culminates in massive expression of p16^INK4a^ [42]. This increase in the levels of p16^INK4a^ by High risk-HPV pointed to the recommendation of this protein as a surrogate marker of HPV infection [18], [19], [43]. According to one recent study, there is an overexpression of p16^INK4a^ staining pattern, diffuse and strong, in all cervical lesions by HPV infections with high-risk and intermediate risk, HPV16, HPV18, HPV31, HPV33, HPV52 and HPV58. In contrast, in lesions with infection by HPV6 and HPV11 the staining pattern is patchy and weak [44]. Still, as noted in this study, p16 expression can be evidenced in cells that are clearly not dysplastic and, thus, its detection in cervical lesions should not be considered as proof, per se, of high risk HPV infection without consideration of other variables, including HPV detection and the hematoxylin and eosin findings.

In the present study we found high prevalence of HPV16 (67.5%), followed by HPV33 (12%) and HPV35 (3.6%). These data provide important HPV DNA information regarding the potential HPV type prevalence in cervical cancer in Rio de Janeiro, Brazil. Our primary limitation is the relatively small number of specimens among the different invasive cancer stages, thus limiting adequate subgroup analyses. Further, it is clear that other molecular biomarkers such as specific microRNAs may be correlated with clinical stage of cervical cancer [44]–[46]; this study did not address this issue.

In conclusion, the present study revealed several correlations in the stage of invasive cancer of the cervix including the clinical variables of advanced age, multiple pregnancies, and paucity of formal education. Molecular variables positively correlated with FIGO stage included expression of Ki-67 and p16. Finally, about 1/3 of the cervical cancers in our cohort were associated with HPV types not specifically targeted by the current HPV vaccines, suggesting that the inclusion of other HPV genotypes may be useful in helping to reduce the incidence of this disease.
